# Obesity management in the setting of endometrial cancer and hyperplasia: A narrative review

**DOI:** 10.1016/j.gore.2025.101929

**Published:** 2025-08-24

**Authors:** Natalie Moshayedi, Kiran Clair, Alex A. Francoeur

**Affiliations:** a California University of Science and Medicine, Colton, CA, USA; b University of California, Irvine, Irvine, CA, USA

**Keywords:** Endometrial cancer, Obesity, GLP-1, Bariatric surgery, Lifestyle

## Abstract

Obesity is a well-established risk factor for endometrial cancer, driven by chronic inflammation, insulin resistance, and excess estrogen. As the global obesity epidemic continues to worsen, effective weight management plays a crucial role in reducing both incidence and progression. Recent pharmacotherapy advancements, particularly GLP-1 receptor agonists, show promising weight loss effects by modulating appetite and metabolism. Preclinical studies suggest these agents may also influence cancer progression, though further clinical research is needed. Bariatric surgery remains the most effective long-term intervention for severe obesity, offering significant metabolic and hormonal benefits. Weight loss surgery is associated with reduced cancer risk and improvements in molecular markers, though findings on specific biomarkers remain inconsistent. This narrative review explores emerging weight loss interventions, focusing on novel pharmacologic agents and bariatric surgery, and their impact on endometrial cancer risk and outcomes.

## Highlights

• Obesity significantly increases risk and mortality in endometrial cancer and hyperplasia.
• GLP-1 receptor agonists may reduce cancer risk via weight loss and molecular modulation.
• Bariatric surgery improves metabolic markers and lowers endometrial cancer incidence.
• Patient and provider awareness of obesity’s oncologic impact remains critically low.
• Integrating obesity management into cancer care may improve outcomes and survivorship.

## 1 Introduction

Endometrial cancer is the only U.S. cancer with declining survival rates over the past four decades and now ranks as the fourth most commonly diagnosed cancer in biological females, with the fastest-growing mortality rate (Siegel et al., 2024). Most cases are detected at an early stage and have a cure rate of 95 % with a combination of surgery and adjuvant chemotherapy or radiation. However, as surgical management typically involves hysterectomy, the increase in the rate of endometrial cancer in young, reproductive aged females has a profound impact on future fertility (Kailasam, 2023). Obesity is well established to be a major risk factor for endometrial cancer (Lu and Broaddus, 2020). It is also associated with an increased risk of death in multiple cancers with the association between endometrial cancer and risk of death being the highest of all cancers affecting biological females (Calle, 2003). Obesity is endemic in the United States with almost 2/3 of American women classified as overweight or obese (Hales, C.M., S. National Center for Health, Prevalence of obesity and severe obesity among adults: United States, 2020).

Addressing this growing public health crisis requires a multifaceted approach that extends across medical and surgical subspecialities. Lifestyle changes like diet and exercise, leading to sustained weight loss, may help reduce risk (Fader et al., 2016). Pharmacologic strategies, such as hormonal therapies and medications targeting insulin resistance, and surgical options offer additional risk-reduction opportunities (Pati, 2023). However, these interventions remain underutilized, and gaps persist in their application to high-risk populations including patients with endometrial hyperplasia and cancer (Luo, 2017). This review aims to summarize and synthesize recent literature regarding the role of lifestyle, medical, and surgical interventions in reducing endometrial cancer risk attributable to obesity to aid clinicians in their management and counseling of these patients.

## 2 Methods

This narrative review explores the link between weight management and endometrial cancer risk and outcomes. A comprehensive PubMed search identified studies on lifestyle changes, hormonal and weight-loss medications, and bariatric surgery effects on endometrial cancer from 1997 to 2025. Priority was given to recent peer-reviewed articles, clinical trials, and large cohort studies. Key findings were synthesized to highlight current evidence, trends, and gaps. No formal *meta*-analysis was done; conclusions were based on qualitative assessment.

### 2.1 Pathophysiology of endometrial cancer and obesity

**Fig. 1:**
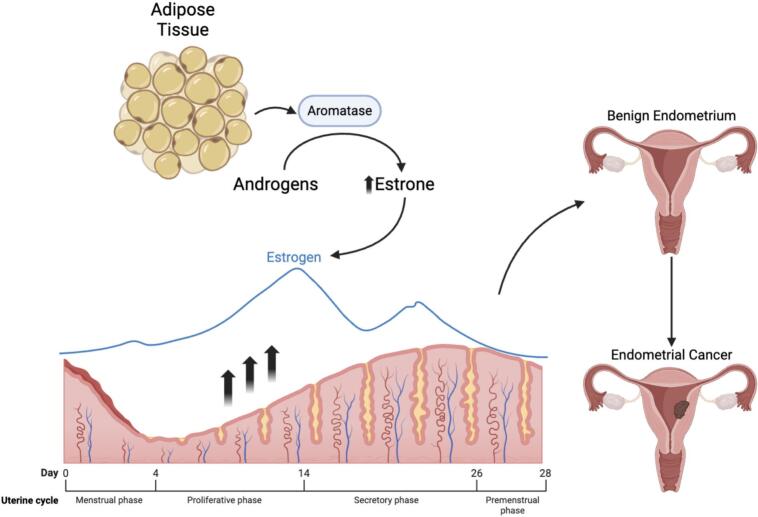
Mechanism of Increased Adiposity on Endometrial Proliferation.

The link between excess weight and endometrial cancer is multifactorial, mainly explained by the unopposed estrogen hypothesis. In premenopausal patients, ovarian granulosa cells produce estrogen driving endometrial proliferation. After menopause, adipose tissue, via aromatase, converts androgens to estrogen, becoming the main estrogen source (Blakemore and Naftolin, 2016, Davis, 2015). Increased adipose tissue and aromatase raise estrogen levels, promoting endometrial proliferation, hyperplasia, and potential malignancy (Fig. 1).

### 2.2 Epidemiology and link between cancer and obesity

The biological link between obesity and endometrial cancer has been consistently observed across epidemiologic studies. A 2003 review highlighted the association between obesity and increased cancer risk and mortality. Specifically, patients with endometrial cancer with a BMI in the obese range (30–34.9 kg/m^2^) experienced a 153 % increase in mortality risk (RR 2.53, 95 % CI 2.02–3.18), while morbidly obese females (BMI ≥ 40 kg/m^2^) faced a 525 % increase in mortality risk due to cancer (RR 6.25, 95 % CI 3.75–10.4) compared to their non-obese counterparts (Calle, 2003). As obesity rates have risen, so has endometrial cancer especially in younger populations as suggested by a 2021 review. Between 1988 and 2016, obesity in women increased from 27 % to 41 %, while the median age of diagnosis dropped from 64.1 to 61.0 years with the most significant rise in women aged 35–44 (Smrz, 2021). A 2014 study projected an endometrial cancer incidence rate of 42.13 cases per 100,000 by 2030, representing a 55 % increase from 2010 rates, largely driven by the escalating obesity epidemic (Sheikh, 2014, Francoeur, 2025). These findings highlight obesity’s role in the rising burden of endometrial cancer in the United States, a trend likely to worsen as obesity rates continue to grow.

Environmental factors can worsen obesity’s impact on endometrial cancer. In a study of over 40,000 gynecologic cancer patients, living in a food desert, defined as an area with limited access to affordable, healthy food, especially fresh fruits and vegetables, was linked to a 43 % higher disease-specific mortality in endometrial cancer (HR 1.43, p < 0.001; 95 % CI 1.22–1.68) (Soliman, 2008). Racial and ethnic disparities in healthcare further complicate the picture. A cross-sectional study using Medical Expenditure Panel Survey data found that Asian (aOR 0.36, 95 % CI 0.16–0.77, p < 0.01), Black (aOR 0.51, 95 % CI 0.39–0.68, p < 0.001), and Hispanic individuals (aOR 0.70, 95 % CI 0.49–0.98, p = 0.04) were significantly less likely to utilize FDA-approved obesity-management medications compared to White individuals, even after adjusting for various factors including socioeconomic status, obesity class, and insurance status (Tagai, 2023). However, it is unknown whether this was due to these populations being offered medical therapy at a lower rate compared to their White counterparts. These findings reflect broader healthcare inequities that may worsen outcomes for minority populations.

Beyond cancer risk, obesity is linked to chronic conditions that further influence endometrial cancer risk. A 2019 *meta*-analysis of 22 studies demonstrated that diabetes as a comorbidity increases the risk of endometrial cancer by 62 % (HR 1.62, 95 % CI 1.34–1.97) (Saed, 2019). Beyond heightened risk of endometrial cancer, female patients with diabetes often experience poorer outcomes, including reduced cancer-specific survival following diagnosis. When accounting for BMI, there was no difference in survival based on diabetes status. This suggests that the association between diabetes and survival is likely attributable to the impact of increased BMI rather than diabetes itself (McVicker, 2022). Polycystic ovary syndrome (PCOS) is another significant risk factor, carrying a 2.79-fold increased risk of developing endometrial cancer (OR 2.79, 95 % CI 1.31–5.95). Among females under 54 years of age, the risk becomes even more pronounced (OR 4.05, 95 % CI 2.42–6.76) (Barry et al., 2014). Higher BMI increases risk of developing PCOS which in turn is associated with a higher risk of endometrial cancer, potentially due to excess estrogen and other BMI related factors (Johnson, 2023). As obesity and related conditions rise in younger people, so does their endometrial cancer risk.

### 2.3 Awareness regarding the link between endometrial cancer and obesity

Despite the known link, public awareness of the cancer risk from excess weight remains limited. A 2008 survey revealed that 58 % of healthy women were unaware that obesity increases the risk of endometrial cancer (Soliman, 2008). Further, a 2023 study focusing on patients with a history of endometrial cancer found that only 53.0 % were aware of the link between their weight and endometrial cancer risk. Notably, only 46 % of participants in this study reported that their doctor had emphasized the importance of maintaining a healthy weight (Tagai, 2023). In another survey, only 29 % of endometrial cancer patients reported being informed by their healthcare providers about the connection between obesity and their condition. Those who were counseled about this link were significantly more likely to attempt weight loss than those who were not informed (p < 0.001), and 56 % of patients who were counseled made dietary changes within 6 months of diagnosis (Clark, 2016). Furthermore, individuals who were aware that obesity is a risk factor for developing endometrial cancer were significantly more likely to perceive weight loss as important compared to those who were not aware that obesity is a risk factor (*β* = 0.248, p = 0.030) (Tagai, 2023). These findings reveal a key gap in patient education, calling for stronger healthcare action. Obesity-related cancer risks should be addressed in primary care and gynecologic visits, with gynecologic oncologists providing counseling and referrals for weight management support.

Another concern is patients’ willingness to accept weight loss counseling during cancer diagnosis and treatment. In a 2015 prospective cohort study, 91 % of patients found it acceptable to discuss weight loss, with 43 % agreeing to a referral to a weight loss specialist, and 12 % accepting a surgical referral. Notably, the interval between hysterectomy and referral for bariatric surgery was found to be a significant predictor of referral acceptance in this study, demonstrating that a new cancer diagnosis is a critical time for change for many patients (Jernigan, 2015). Approximately 51 % of patients in this cohort were interested in a formal weight loss program and 85 % of endometrial cancer survivors would prefer their gynecologic oncologist to address weight using face-to-face counseling with specific recommendations including referral to specialists (Tagai, 2023, Tseng, 2015).

While patient education is vital, physicians must also stay informed on novel obesity management strategies. A survey of 454 gynecologic oncology providers revealed that only 10 % of these providers had received formal training in weight loss counselling (Neff, 2014). In a survey of clinicians, both primary care physicians (84.5 %) and gynecologic oncologists (85.1 %) reported that they addressed the role obesity plays in cancer risk with their patients (Jernigan, 2013). Furthermore, a survey of bariatric surgery providers found that only 21 % referred patients for endometrial evaluations, and just 20 % of surgeons routinely counsel patients on the increased cancer risks associated with obesity (Winfree, 2010).

These studies show that although clinicians know the obesity–endometrial cancer link, they often avoid discussing weight with patients. However, patients are receptive to counseling and a cancer diagnosis could be a critical moment to provide resources and referrals patients can act on. Additionally, as gynecologic oncologists manage a large proportion of obesity related cancer, increased obesity medicine training or collaboration could be of benefit. More cancer care is being housed in comprehensive cancer centers across the United States where patients benefit from seeing their oncologist, a social worker, and even palliative medicine. Obesity medicine could also be incorporated into a holistic cancer care model.

## 3 Studies investigating lifestyle interventions to address cancer risk

### 3.1 Diet and supplement-based interventions

Diet and food intake has been extensively studied in relation to obesity. Cohen et al. examined the impact of a ketogenic diet on central obesity and serum insulin levels, demonstrating that it decreased fat mass while preserving lean body mass. They also found that this diet improved insulin sensitivity and increased levels of beta-hydroxybutyrate, a compound previously shown to inhibit cancer cell proliferation (Cohen, 2018). Additionally, the researchers assessed quality of life factors, finding that the ketogenic diet improved physical function using the Medical Outcomes Study Short Form-12 Health Survey, increased energy levels, and reduced specific food cravings in patients with endometrial cancer (Cohen, 2018). Counseling regarding ketogenic based diets could be an intervention for patients with endometrial cancer and patients could benefit from routine referral to a nutritionist or dietician.

Retrospective studies have assessed specific food intake associated with endometrial cancer. Generally, researchers found that increased fat intake was associated with higher risk, while fiber was inversely related to endometrial cancer risk. Further, soy, legumes, whole grains, vegetables, fruits, and seaweeds were inversely related to cancer risk (Goodman, 1997). Red meat conferred a twofold increased cancer risk with just one increased serving per week (OR 2.07, 95 % CI 1.29–3.33) while decreased risk was observed for coffee (OR 0.83, 95 % CI 0.72–0.95) and vegetables (OR 0.83, 95 % CI 0.72–0.95) (Bravi, 2009). In addition, a specific diabetes risk reduction diet (DRRD) which included high proportions of fiber, fruit, and coffee and lower proportions of red meat decreased endometrial cancer risk (OR 0.45, 95 % CI 0.28–0.73) (Esposito, 2021).

Delivery of weight loss counseling via technology-based programs have garnered recent research interest. One study compared a telemedicine platform and a text messaging intervention aimed at facilitating weight loss while also monitoring biomarkers before and after treatment. Both interventions promoted weight loss, but the telemedicine platform proved more effective with a greater decrease in weight (Haggerty, 2016). A later study explored a similar idea comparing a text-message intervention and standard care. In this case, the text message-based intervention did not help with weight loss in obese patients with endometrial cancer (Zamorano, 2021). These two studies indicate that face-to-face interventions even through telemedicine can improve weight loss in this population.

Researchers have also investigated the link between diet and age of menopause as late menopause is a risk factor for endometrial cancer. People who eat a plant-based diet have been shown to have an earlier age of menopause while high consumptions of meat and fat have been associated with a later onset of menopause (Dunneram, 2018). Other studies found that higher intake of green and yellow vegetables was associated with earlier menopause, whereas higher intake of certain dairy products and lower alcohol intake were associated with later menopause; however, overall evidence remains inconsistent and more research is needed to clarify these associations (Grisotto, 2022) (Table 1).

**Table 1:** Summary of Diet and Supplement Based Interventions.

Study	Study Design	Intervention	Primary Outcome(s)	Key Findings
Cohen et al.	Prospective cohort	Ketogenic diet	Central obesity, insulin sensitivity, beta-hydroxybutyrate levels, quality of life	Decreased fat mass, preserved lean mass, improved insulin sensitivity, increased beta-hydroxybutyrate, improved physical function, and reduced food cravings.
1997 Dietary Study	Observational	Dietary intake analysis	Endometrial cancer risk	High fat intake increased cancer risk; fiber, soy, legumes, whole grains, vegetables, fruits, and seaweeds were inversely related to risk.
2009 Dietary Study	Case-control	Dietary patterns (red meat, coffee, cereals, vegetables)	Endometrial cancer risk	Red meat increased risk (OR 2.07, 95 % CI 1.29–3.33), while coffee (OR 0.83, 95 % CI 0.72–0.95) and vegetables (OR 0.83, 95 % CI 0.72–0.95) decreased risk.
2021 Diabetes Risk Reduction Diet (DRRD) Study	Prospective cohort	High fiber, fruit, coffee, low red meat diet	Endometrial cancer risk	DRRD significantly reduced cancer risk (OR 0.45, 95 % CI 0.28–0.73).
Telemedicine vs. Text Messaging Study	Randomized controlled trial	Telemedicine vs. text messaging for weight loss	Weight loss	Both interventions promoted weight loss
Zamorano et al.	Randomized controlled trial	Text messaging vs. standard care	Weight loss in obese endometrial cancer patients	Text messaging intervention did not improve weight loss.
Diet and Menopause Timing Study	Observational	Plant-based diet vs. high meat/fat diet	Age of menopause	Plant-based diets linked to earlier menopause; high meat/fat intake associated with later menopause. Potential implications for endometrial cancer risk due to prolonged estrogen exposure.

### 3.2 Physical activity-based interventions

Exercise plays a role in many lifestyle interventions looking to address weight loss. A 2010 study showed that patients who were active throughout their lifetime had decreased risk of endometrial cancer (OR 0.61, 95 % CI 0.43–0.87) (John et al., 2010). Despite this association, lifestyle changes are often challenging to initiate and even harder to maintain. In a 2015 survey of endometrial cancer survivors, patients with increased body mass index reported significantly decreased exercise frequency (p = 0.016) (Tseng, 2015). Another 2015 study also showed that only 14.2 % of endometrial cancer patients were able to exercise without supervision based on their health status at the time of diagnosis (Zhang, 2015). To assess feasibility and efficacy in exercise interventions, a 2022 study employed individualized exercise intervention in endometrial cancer patients after treatment. They identified a significant improvement in visceral fat percentage (p = 0.039) and physical fitness (six-minute walk test, p < 0.001) and showed a maximum weight loss of 6.0 kg after 3 months and 8.4 kg after 6 months (Smits, 2022).

Although evidence supports that lifestyle changes, including weight loss interventions, can improve outcomes for endometrial cancer, von Grueningen et al. investigated the feasibility of such interventions. Researchers implemented a six-month lifestyle program incorporating individual counseling on nutrition and exercise. The study demonstrated that lifestyle intervention programs are feasible and can lead to sustained weight loss and behavioral changes over a year (von Gruenigen, 2008). In addition to the studies mentioned previously, there is ongoing work currently investigating how best to approach lifestyle changes in conjunction with uterine conserving medical management in the treatment and prevention of endometrial cancer (NCT05903131). These findings highlight the potential of structured lifestyle programs to serve as an adjunctive approach in reducing risk factors and improving long-term outcomes for patients with endometrial cancer.

## 4 Pharmaceutical based interventions for weight loss and cancer risk

### 4.1 Metformin

The potential role of pharmaceutical weight loss interventions in the management of endometrial hyperplasia and cancer has been extensively studied, with a large amount of the literature examining the links between metformin use, weight loss, and cancer risk. Commonly used in patients with diabetes to enhance insulin sensitivity, metformin has also been investigated for its potential effects on weight loss and cancer prevention. While retrospective studies have suggested a potential role for metformin in reducing endometrial proliferation and cancer risk, prospective trials have shown mixed results. Early studies such as Pabona et al. and Petchilisa et al. indicated reductions in tumor biomarkers and cell proliferation markers (Pabona, 2020, Petchsila, 2020). However, the PREMIUM trial, a 2018 multicenter, double-blind study, found no significant change in cell proliferation with metformin compared to placebo (Kitson, 2019). Prospective studies such as Yates et al. showed mixed results. These researchers compared metformin and lifestyle interventions, and while lifestyle intervention produced the most significant weight loss, neither treatment altered biomarkers or endometrial proliferation after 16 weeks of treatment (Yates, 2018). Recently, Konstantinopoulos et al. studied metformin’s effect on estrogen-receptor positive endometrial cancer with endometroid histology. They noted that metformin added to letrozole/abemaciclib is feasible and safe and appears to induce improved responses and prolonged progression free survival than letrozole/abemaciclib alone, a promising finding (Konstantinopoulos, 2025).

### 4.2 Incorporation of progesterone-based therapeutics with metformin

Progesterone intrauterine devices (IUDs), such as those releasing levonorgestrel, are commonly utilized for their localized delivery of progestin to the endometrium, effectively reversing hyperplasia and offering a non-surgical treatment option. The therapeutic benefits of levonorgestrel IUDs have also prompted investigations into combining this modality with adjunct therapies, such as metformin. It is important to note that standard of care recommendations for complex atypical hyperplasia (CAH) endometrial cancer is for hysterectomy. However, given the previously discussed rising rate of patients diagnosed at an reproductive age with these conditions, conservative management options have garnered significant interest and have demonstrated success in these populations (Westin, 2021).

To enhance treatment efficacy, adjunctive therapies such as metformin have been explored due to their potential antiproliferative and weight loss effects. However, while retrospective studies report conflicting outcomes, several prospective trials have consistently demonstrated that the primary driver of regression is likely progestin-based therapy (Ravi, 2021). Adjunctive metformin may contribute to weight loss which has been associated with improved response rates, but metformin alone has not improved histologic outcomes when added to progestin therapy (Janda, 2021, Mitsuhashi, 2016). Some data suggest that the combination of metformin with oral or intrauterine progestins may modestly reduce relapse in CAH and improve outcomes in non-atypical hyperplasia, though findings in endometrial cancer remain inconsistent (Yang, 2020, Tehranian, 2021).

A 2024 Cochrane review compared metformin use to megestrol, metformin and megestrol with megestrol alone, and metformin plus levonorgestrel (intrauterine system) and levonorgestrel (intrauterine system) alone in endometrial hyperplasia regression. In this comparison, metformin increased rate of regression to 71–96 % and metformin along with megestrol increased rate to 66–84 % while megestrol alone was 61 %. The use of intrauterine levonorgestrel alone showed that the rate of regression was 96 % and the addition of metformin changed the rate of regression to be between 73–100 % (Shiwani, 2024). Although no significant difference was observed, further research may be needed to explore the long-term effects of metformin on endometrial proliferation. The concurrent usage of metformin and progesterone has improved responses in endometrial hyperplasia than metformin alone and could be considered as adjunctive treatment in addition to progesterone-based therapy for CAH or early-stage endometrial cancer desiring fertility sparing management (Table 2). These results continue to be verified and studied through new clinical trials such as NCT02035787, a phase 2 study examining the addition of metformin to a levonorgestrel IUD for CAH or clinical stage 1 endometrial cancer.

**Table 2:** Summary of Biomarkers Metformin-Based Interventions.

Study	Study Design	Intervention	Primary Outcome(s)	Key Findings
Pabona et al.	Observational	Metformin vs. no metformin	Expression of tumor biomarkers (Ki67, TUNEL, ERα, PR, PTEN, KLF9)	Metformin altered steroid receptor expression and key signaling pathways, suggesting potential anticancer effects.
Petchilisa et al.	Prospective cohort	Short-term metformin therapy	Ki67 proliferation index	Significant reduction in Ki67 levels, supporting metformin’s antiproliferative effects.
PREMIUM Trial (2018)	Multicenter, double-blind RCT	Metformin vs. placebo for 1–5 weeks preoperatively	Ki67 proliferation index	No significant reduction in Ki67 with metformin treatment, questioning its antitumor efficacy.
Yates et al.	Randomized controlled trial	Metformin vs. lifestyle intervention	Weight loss, endometrial biomarkers, proliferation	Lifestyle intervention led to the most significant weight loss; neither intervention significantly altered biomarkers or proliferation at 16 weeks.
2024 Cochrane Review	Systematic review and meta-analysis	Metformin ± megestrol vs. levonorgestrel IUD	Regression of endometrial hyperplasia	Regression rates: metformin (71–96 %), metformin + megestrol (66–84 %), megestrol alone (61 %), levonorgestrel IUD (96 %), levonorgestrel IUD + metformin (73–100 %).
Janda et al. (2021)	Prospective study	Metformin + levonorgestrel IUD	Pathological complete response, weight loss	Metformin led to significant weight loss but decreased response rates in endometrial cancer.
Phase II Study	Clinical trial	Medroxyprogesterone acetate + metformin	Disease relapse in complex atypical hyperplasia (CAH) and endometrial cancer	Metformin reduced relapse rates from 30–50 % to 6.4 %.
Yang et al.	Randomized controlled trial (China)	Metformin + megestrol vs. megestrol alone	Response in CAH and endometrial cancer	Combination therapy improved hyperplasia response but did not significantly impact cancer outcomes (OR 0.58, 95 % CI 0.07–5.11, p = 0.63).
Tehranian et al.	Prospective study	Metformin + megestrol	Treatment response in non-atypical endometrial hyperplasia	Addition of metformin significantly improved treatment response rates.
Ravi et al.	Prospective cohort	Metformin + levonorgestrel IUD	Endometrial hyperplasia regression, BMI	No significant impact on hyperplasia regression, but BMI decreased.
Konstantinopoulos et al.	Phase 2 RCT	Metformin + Letrozole/Abemaciclib	ER + endometroid endometrial cancer	Increased progression-free survival as well as showed a stronger response than Letrozole/Abemaciclib alone.

### 4.3 Novel weight loss agents

While metformin has been used for weight management for decades, newer therapies such as GLP-1 receptor agonists are emerging as promising alternatives. Given the recent enthusiasm for GLP-1 inhibitors, particularly for weight loss, their effects on the endometrium warrant further study (Ghusn, 2022). Though the exact mechanism for weight loss is unknown, GLP-1 inhibitors have been shown to decrease gastric emptying, reduce feelings of hunger, and increase measures of satiety directly in the hypothalamus (Ard, 2021). GLP-1 is expressed in various tissues in the body and there is concern that the medication could increase cancer risk (Elashoff, 2011). Several meta-analyses have not found an association with GLP-1 agonist use and cancer risk but continued data monitoring is important (Sun, 2024, Liu, 2019).

Preclinical studies by Zhang et al. using nude rats showed that the GLP-1 agonist exenatide could attenuate endometrial cancer growth (Zhang, 2016). In another study, Kanda et al. found that higher GLP-1R expression in human endometrial cancer cell lines treated with liraglutide, a GLP-1 agonist, was significantly correlated with better progression-free survival (Kanda, 2018). A 2025 study investigated the combination of levonorgestrel and semaglutide in cell models. Their findings suggest that due to the synergistic interaction between GLP-1R and steroid hormone receptor pathways, semaglutide may enhance the anticancer effects of levonorgestrel (Hagemann, 2025). Notably, a 2025 meta-analysis found that there was a significant reduction in uterine cancer risk among obese patients with the use of these medications (Silverii, 2025). As this research is relatively new compared to studies on metformin, ongoing studies are assessing the effects of the GLP-1 agonist tirzepatide on both endometrial cancer and endometrial atypia (NCT06073184).

### 4.4 Surgical interventions for weight loss and cancer risk

Bariatric surgery is a well-established treatment for severe obesity, offering significant and sustained weight loss as well as metabolic and hormonal changes. Bariatric surgery encompasses various surgical techniques, such as Roux-en-Y gastric bypass, sleeve gastrectomy, and adjustable gastric banding, which alter the gastrointestinal anatomy to reduce caloric intake and, in some cases, nutrient absorption. These changes have been shown to mitigate the risk of obesity-related conditions, including endometrial cancer, which is strongly associated with hyperestrogenism, chronic inflammation, and insulin resistance. Emerging evidence suggests that bariatric surgery may modify these pathophysiological mechanisms, potentially reducing the risk and progression of endometrial cancer (Ross, 2022).

Prospective studies have investigated the impact of bariatric surgery on endometrial cancer, including patients with and without pre-existing endometrial cancer. Some findings showed that weight loss surgery led to decreased molecular expression of markers of endometrial proliferation, while reducing insulin resistance and increasing in reproductive biomarkers (MacKintosh, 2019, Linkov, 2017) This demonstrates a biologic basis for weight loss surgery being an efficacious adjunctive therapy for endometrial cancer. However, these findings were not supported as strongly in follow up studies with respect to endometrial proliferation after bariatric surgery (Linkov, 2014, Argenta, 2014) (Table 3).

**Table 3:** Summary of Biomarkers in Prospective Surgical Interventions.

Biomarker	C-peptide	CRP	IL-1Rα	IL-6	Insulin	Leptin
Linkov (2017)	Decreased*	Decreased*	Decreased*	Decreased*	Decreased*	Decreased*

	**CD3**	**CD20**	**PTEN**			
Linkov (2014)	No significant change	No significant change	No significant change			

	**Ki67**	**CRP**	**ER/PR**	**IL-6**	**Insulin**	**Leptin**
Mackintosh	Decreased*	Decreased*	Decreased*	Decreased*	Decreased*	Decreased*

	**Ki-67**	**ER**	**PR**	**AR**		
Argenta	No significant change	Decreased*	Decreased*	Decreased		

*p < 0.05.

Despite mixed results of prospective studies, retrospective cohort studies overall showed decreased risk of endometrial cancer in patients who received bariatric surgery. A few addressed the impact of bariatric surgery on the risk of endometrial cancer on a population level. One study found that undergoing bariatric surgery reduced the risk of developing endometrial cancer by one half with a HR of 0.50 (95 % CI 0.37–0.67) (Schauer, 2019). Other studies globally confirmed this decreased risk with a reported HR of 0.56 (95 % CI 0.35–0.89) (Schauer, 2019, Anveden, 2017). In contrast, an all-English cohort study found no decreased risk of endometrial cancer post-obesity surgery likely due to the small sample size (Aravani, 2018).

One case report highlights the potential impact bariatric surgery could have on endometrial cancer. A young woman under 18 years old with endometrial cancer and a BMI of 36.2 experienced failure of fertility-preserving treatment using a levonorgestrel-releasing intrauterine device. Nine months after bariatric surgery and 18 months after IUD insertion, the patient achieved a normal body weight with a BMI of 20.3 and showed a complete response to treatment, suggesting that bariatric surgery may be an effective adjunct treatment for fertility preservation in young endometrial cancer patients (Benito, 2015).

Studies indicate that weight loss surgery can play a significant role in reducing the risk of endometrial cancer and improving treatment outcomes. Neff et al. evaluated the economic implications of providing bariatric surgery to low-risk, morbidly obese (BMI > 40) patients with endometrial cancer. They found that weight loss surgery had an incremental cost-effectiveness ratio of $26,080 per quality-adjusted life year, attributed in part to the improved quality of life associated with weight reduction and decreased cancer risk (Neff, 2015). Additionally, an ongoing clinical trial (NCT04839614) is investigating the feasibility of combining bariatric surgery with hysterectomy in patients with endometrial cancer to optimize disease outcomes and quality of life. Based on these findings, physicians managing young, obese patients with endometrial cancer or hyperplasia should consider referral to bariatric surgeons as part of a multidisciplinary approach to care.

### 4.5 Strengths and limitations

The emerging evidence surrounding weight loss interventions for endometrial cancer highlights several strengths. Novel agents such as GLP-1 receptor agonists show promise in achieving significant weight loss. Additionally, bariatric surgery demonstrates robust and sustained benefits, with substantial reductions in endometrial cancer risk and improvements in biomarkers. These findings are supported by large cohort studies and ongoing clinical trials, which bolster their relevance and applicability in clinical settings.

However, there are some limitations. Much of the research on GLP-1 receptor agonists is early stage, with limited long-term data and a reliance on preclinical studies and small sample sizes. Similarly, bariatric surgery studies show variability with conflicting results from short-term cohorts. The impact of obesity surgery on endometrial cancer risk remains inconclusive in some studies, emphasizing the need for standardized methodologies. Economic and access barriers to both treatments persist, especially in underserved populations. Future research should expand sample sizes, explore long-term outcomes, and address health disparities to improve generalizability.

## 5 Future directions

Future research should prioritize long-term evaluation of GLP-1 receptor agonists and their effects on endometrial cancer prevention and progression, with a focus on mechanisms of action and patient outcomes. Clinical trials exploring agents like tirzepatide offer a promising avenue to understand the interplay between weight loss, metabolic changes, and endometrial remodeling. Integrating advanced molecular profiling into bariatric surgery studies could elucidate biological pathways behind reduced cancer risk, including roles of hormonal regulation, inflammation, and insulin resistance.

Another key area is developing personalized treatment strategies for obese endometrial cancer patients. This includes assessing the efficacy of combining bariatric surgery with curative interventions like hysterectomy and optimizing fertility-preserving treatments in younger populations. Developing risk-based scoring tools would help stratify patients at risk for treatment failure or recurrence. With better predictive models, aggressive obesity interventions can be more individualized. Addressing disparities in access to weight loss treatments is also critical to ensure diverse patient benefit. Economic analyses should continue evaluating the broader impact of these therapies on cancer management, informing policy and healthcare allocation.

## 6 Conclusions

Emerging weight management therapies, including GLP-1 receptor agonists and bariatric surgery, show strong potential in reducing endometrial cancer risk and improving outcomes. GLP-1 agonists may slow cancer growth and enhance survival, while bariatric surgery is linked to reduced cancer risk and favorable changes in obesity-related biomarkers. Despite promising findings, further research is needed to understand long-term outcomes and refine treatment for specific populations. Given racial and ethnic disparities in endometrial cancer, more studies should explore differences in access to obesity medications and surgical options. Incorporating weight management into care plans for obese endometrial cancer patients could improve survival and quality of life.

## CRediT authorship contribution statement

**Natalie Moshayedi:** Writing – review & editing, Writing – original draft, Formal analysis, Conceptualization. **Kiran Clair:** Writing – review & editing, Supervision. **Alex A. Francoeur:** Writing – review & editing, Formal analysis, Conceptualization.

## Declaration of Competing Interest

The authors declare that they have no known competing financial interests or personal relationships that could have appeared to influence the work reported in this paper.
